# Modulations of Histone Deacetylase 2 Offer a Protective Effect through the Mitochondrial Apoptosis Pathway in Acute Liver Failure

**DOI:** 10.1155/2019/8173016

**Published:** 2019-04-28

**Authors:** Yao Wang, Fan Yang, Fang-Zhou Jiao, Qian Chen, Wen-Bin Zhang, Lu-Wen Wang, Zuo-Jiong Gong

**Affiliations:** Department of Infectious Diseases, Renmin Hospital of Wuhan University, Wuhan, China

## Abstract

The purpose of this study was to investigate the modulation of histone deacetylase 2 (HDAC2) on mitochondrial apoptosis in acute liver failure (ALF). The cellular model was established with LO2 cells stimulated by tumor necrosis factor alpha (TNF-*α*)/D-galactosamine (D-gal). Rats were administrated by lipopolysaccharide (LPS)/D-gal as animal model. The cell and animal models were then treated by HDAC2 inhibitor CAY10683. HDAC2 was regulated up or down by lentiviral vector transfection in LO2 cells. The mRNA levels of bcl2 and bax were detected by real-time PCR. The protein levels of HDAC2, bcl2, bax, cytochrome c (cyt c) in mitochondrion and cytosol, apoptosis protease activating factor 1 (apaf1), caspase 3, cleaved-caspase 3, caspase 9, cleaved-caspase 9, acetylated histone H3 (AH3), and histone H3 (H3) were assayed by western blot. Apoptosis was detected by flow cytometry. The serum alanine aminotransferase (ALT), aspartate aminotransferase (AST), and total bilirubin (TBIL) levels were also assayed. The openness degree of the mitochondrial permeability transition pore (MPTP) was detected by ultraviolet spectrophotometry. The apoptosis of hepatocytes in liver tissues was determined by tunnel staining. The liver tissue pathology was detected by hematoxylin eosin (HE) staining. The ultrastructure of liver tissue was observed by electron microscopy. Compared with cell and rat model groups, the bax mRNA level was decreased, and bcl2 mRNA was increased in the CAY10683 treatment group. The protein levels of HDAC2, bax, cyt c in cytosol, apaf1, cleaved-caspase 3, and cleaved-caspase 9 were decreased, and the apoptosis rate was decreased (*P* < 0.05), whereas the protein level of bcl2 and cyt c in the mitochondrion was elevated (*P* < 0.05) in the CAY10683 treatment group. In the HDAC2 down- or upregulated LO2 cells, the mitochondrial apoptosis pathway was inhibited or activated, respectively. After being treated with TNF-*α*/D-gal in HDAC2 down- or upregulated LO2 cells, the mitochondrial apoptosis pathway was further suppressed or activated, respectively. The MPTP value was elevated in CAY10683-treated groups compared with the rat model group (*P* < 0.05). Liver tissue pathological damage and apoptotic index in the CAY10683-treated group were significantly reduced. In addition, AH3 was elevated in both cell and animal model groups (*P* < 0.05). Downregulated or overexpressed HDAC2 could accordingly increase or decrease the AH3 level, and TNF-*α*/D-gal could enhance the acetylation effect. These results suggested that modulations of histone deacetylase 2 offer a protective effect through the mitochondrial apoptosis pathway in acute liver failure.

## 1. Introduction

Acute liver failure (ALF) is an extremely serious clinical syndrome caused by various factors. Its pathological characteristic shows that there is necrosis in a large number of hepatocytes and inflammatory cell infiltration in the liver. ALF has features of acute onset, quick development, serious complications, and high mortality [1]. Previous studies have verified that intestinal endotoxemia plays an important role during development of ALF. Lipopolysaccharide (LPS), the main component of endotoxin, induces liver microcirculation disorder and causes serious damages in liver cells [2]. Endotoxin can also stimulate Kupffer cells to release many cytokines such as TNF-*α*, IL-1, and IL-6, which further result in liver necrosis [3].

As a common form of cell death, apoptosis contributed to liver injury in a wide range of acute and chronic liver diseases. In liver disease, hepatocytes would die through the apoptosis pathway [4]. Apoptosis is usually induced in two ways. One is the death receptor/extrinsic pathway; another is the mitochondrial/intrinsic pathway. The former can be activated by combining death signal mediators, such as TNF-*α*, with death receptors on the cell surface. The latter manner can be activated by cytochrome c released from mitochondria [5, 6]. These two proapoptotic signals can be activated by caspase 8 and caspase 9, which further result in the activation of caspase 3, cleaving of substrate proteins, destruction of DNA molecules, and cell apoptosis [7]. Because hepatocytes are enriched with mitochondria, liver is the chemical center of the human body and accounts for 20% of the total body oxygen consumption [8]. Therefore, the mitochondrial apoptotic pathway plays an important role in hepatocellular apoptosis [9]. It is important to investigate hepatocyte mitochondrial apoptosis in ALF.

Acetylation regulation involving histone acetyltransferases (HATs) and histone deacetylase (HDACs) is important to maintaining homeostasis [10]. HDACs have been verified to regulate homeostasis by inhibiting gene transcription [10, 11]. In the previous study, tumor suppressor breast cancer susceptibility gene 1 (BRCA1) has been found to inhibit miR-155 via its association with HDAC2. HDAC2 deacetylates histones H2A and H3 on the miR-155 promoter [12]. Many studies have also shown that acetylation played a key role in liver injury. HDAC broad-spectrum inhibitor trichostatin A (TSA) can effectively alleviate septic liver injury [13, 14]. Histone deacetylase 4 promotes cholestatic liver injury in the absence of prohibitin-1 [15]. These results are consistent with our previous findings that TSA can effectively inhibit the release of multiple inflammatory factors, improve hepatocyte injury, and improve the survival rate of rats with acute-on-chronic liver failure [16]. The HDAC2 inhibitor CAY10683 achieves the goal of treating acute liver failure by protecting the damaged intestinal mucosa and reducing intestinal endotoxemia [17]. Most of these studies are based on the mechanism association with acetylation in the process of inflammation. However, the direct protective effects of HDACs on hepatocytes through mitochondrial apoptosis during liver failure have been rarely studied.

In this study, we aimed to investigate the effect of HDAC2 on TNF-*α*/D-gal-induced injury in the LO2 cell line and in the LPS/D-gal-induced ALF rat model and to further explore the HDAC2 epigenetic regulation on the mitochondrial apoptosis pathway in acute liver failure.

## 2. Material and Methods

### 2.1. Chemicals and Reagents

HDAC2 inhibitor CAY10683 was purchased from Sellect (Houston, USA). DMEM basic and fetal bovine serum (FBS) were obtained from Gibco (NY, USA). Lipopolysaccharide (LPS, purity of 99%), tumor necrosis factor alpha (TNF-*α*, purity of 97%), and D-galactosamine (D-gal, purity of 98%) were purchased from Sigma (St. Louis, USA). Rabbit anti-rat/human HDAC2, bcl2, cytochrome c (cyt c), apoptosis protease activating factor 1 (apaf1), caspase 3/cleaved-caspase 3, caspase 9, cleaved-caspase 9, histone H3 (H3), and acetylated histone H3 (AH3) antibodies were obtained from Cell Signaling Technology (Boston, USA). Rabbit anti-rat/human bax antibody was obtained from Abcam (Cambridge, UK). Glyceraldehyde-3-phosphate dehydrogenase (GAPDH) and cytochrome c oxidase subunit 4 isoform 1 (cox IV) antibodies were purchased from Proteintech (Wuhan, China). The goat anti-rabbit fluorescent secondary antibody (IRDye800) was obtained from LI-COR Biosciences Inc. (Lincoln, USA). RNAiso Plus, PrimeScript™ RT reagent, and SYBR Premix Ex Taq kits were purchased from TaKaRa (Dalian, China). The purified mitochondrial permeability transition pore (MPTP) fluorescence detection kit was purchased from GENMED (Shanghai, China).

### 2.2. Cell Culture and Chemical Treatment

A DMEM medium mixed with 10% FBS was used to culture LO2 cells at 37°C and saturated humidity and using a 5% CO_2_ incubator. The medium was replaced every 2-3 days. The cells were divided into control, model, and CAY10683-treated groups. TNF-*α* and D-gal were used to establish an *in vitro* model. When the cells were 70% confluent, they were passed into 6-well plates for 24 h. Then, TNF-*α* (100 ng/mL) and D-Gal (44 *μ*g/mL) were used to stimulate the cells except for the control group. CAY10683- (120 nM) treated cells were harvested after a 24-hour incubation.

### 2.3. Knockdown or Overexpression of HDAC2 in LO2 Cells via Lentiviral Transduction

HDAC2 knockdown- or overexpression-lentiviral vectors were constructed by GeneCreate Biological Engineering Co. Ltd. (Wuhan, China). The knockdown interference sequence was 5′-GCAAATACTATGCTGTCAATT-3′ (21 bp). The cloning vectors were transfected into the 293 T cell line, and the viral supernatant was harvested after 48 h (1 × 10^8^ transducing units (TU)/mL). Then, LO2 cells were seeded into 24-well plates and transfected with lentivirus vectors. According to the manufacturer's instructions, the tested multiplicity of infection (MOI) value was 40. After being transfected for 72 h, knockdown or overexpression of HDAC2 was confirmed by RT-PCR analysis.

### 2.4. LO2 Cell Apoptosis Detection

The LO2 cell apoptosis rates were detected by flow cytometry with Annexin V-phycoerythrin/7-aminoactinomycin D (Annexin V-PE/7AAD) apoptosis kit (BD, USA). After the 10^5^ cells were processed to a 6-well plate, they were collected for staining. The cells were added with 400 *μ*L buffer solution, 5 *μ*L Annexin V-PE, and 5 *μ*L 7AAD, then incubated for 15 min at 37°C away from light. Flow cytometry (BD, USA) was adopted to count the apoptosis rate in the early and late stages.

### 2.5. Animal Groups

A total of 30 rats were randomly divided into three groups: control group, model group, and CAY10683-treated group. The model and CAY10683 group rats were administrated by intraperitoneal injection with 400 mg/kg D-gal combined with 100 *μ*g/kg of LPS. CAY10683 and the same amount of saline were given to the CAY10683-treated group and model group 2 h before ALF modeling was conducted. All rats were sacrificed under anesthesia for 48 h.

### 2.6. Specimen Collection, Histological Studies, and Biochemical Tests

Fresh liver specimens were fixed in 10% neutral-buffered formalin for 2 days and then processed for sectioning and staining by standard histological methods. Sections from the liver were stained with H&E and evaluated under light microscope (Olympus, Tokyo, Japan). Liver samples were fixed by 2.5% glutaraldehyde and 1% glutaric acid. After dehydration by ethanol and acetone, epoxy resin was embedded and sliced, and saturated uranium acetate and lead citrate were stained double. The serum alanine aminotransferase (ALT), aspartate aminotransferase (AST), and total bilirubin (TBIL) levels were assayed using a fully automated Aeroset chemistry analyzer (Abbott Co. Ltd., USA).

### 2.7. Quantitative Real-Time PCR to Detect mRNA Expression

The PCR procedure followed the previously published steps [18]. Total RNAs from LO2 cells and liver specimens were isolated by using RNAiso Plus according to the manufacturer's protocol. The cDNAs were produced with a PrimeScript RT reagent kit and incubated at 37°C for 15 min and at 85°C for 5 s. Quantitative real-time PCRs were performed using a StepOnePlus device (Applied Biosystems) at 95°C for 10 s, followed by 40 cycles at 95°C for 5 s and at 60°C for 20 s, according to the instructions for the SYBR Premix Ex Taq kit. The data were analyzed by the 2^−ΔΔCT^ method. All the primer sequences (Table 1) were designed and synthesized by Tsingke (Wuhan, China). GAPDH was set as the housekeeping gene.

### 2.8. Western Blotting for Protein Expression Measurement

Isolation of proteins in the mitochondrion and cytosol were performed following the method described by Cai et al. [19]. Proteins from the LO2 cell, liver specimen, mitochondrion, and cytosol were separated by SDS-PAGE gels and then transferred onto a nitrocellulose filter membrane, respectively. The membranes were blocked overnight with 5% nonfat milk in phosphate-buffered saline (PBS) and probed with the indicated antibody (Ab) before being washed three times in Tris-buffered saline with Tween 20 (TBST) and then incubated with an HRP-labeled secondary Ab. The dilutions of the primary and secondary antibodies were as follows: HDAC2, 1 : 1000; bcl2, 1 : 1000; cyt c, 1 : 1000; apaf1, 1 : 1000; caspase 3/cleaved-caspase 3, 1 : 1000; caspase 9, 1 : 1000; cleaved-caspase 9, 1 : 1000; H3, 1 : 1000; AH3, 1 : 1000; and GAPDH, 1 : 1000. Then, they were incubated with a fluorescent secondary antibody at 37°C for 2 h. The blot was analyzed using the Odyssey Infrared Imaging System (LI-COR Biosciences). GAPDH and histone 3 were set as the housekeeping genes.

### 2.9. Relative Fluorescence Unit (RFU) of Mitochondrial MPTP in Hepatocytes

Fluorescence enzyme assay was used to detect bis-(bis-carboxymethyl aminomethyl fluorescein)-acetoxymethyl ester. When the mitochondrial permeability transition pore (MPTP) was in an open state, calcitonin would release out of the mitochondria. The fluorescence changes of calcitonin in mitochondria represented the open state of MPTP. The specific experimental procedures were carried out in accordance with the product specification of the purified mitochondrial membrane channel fluorescence detection kit (GENMED, China).

### 2.10. Statistical Analysis

Data were statistical analyzed with SPSS 13.0 software. The results were expressed as means ± SDs. One-way analysis of variance (ANOVA) or Student's *t* test was performed to examine the differences between groups. A *P* value less than 0.05 was considered statistically significant.

## 3. Results

### 3.1. CAY10683 Inhibited the Mitochondrial Apoptotic Pathway in the TNF-*α*/D-Gal-Treated LO2 Cells

**Figure 1:**
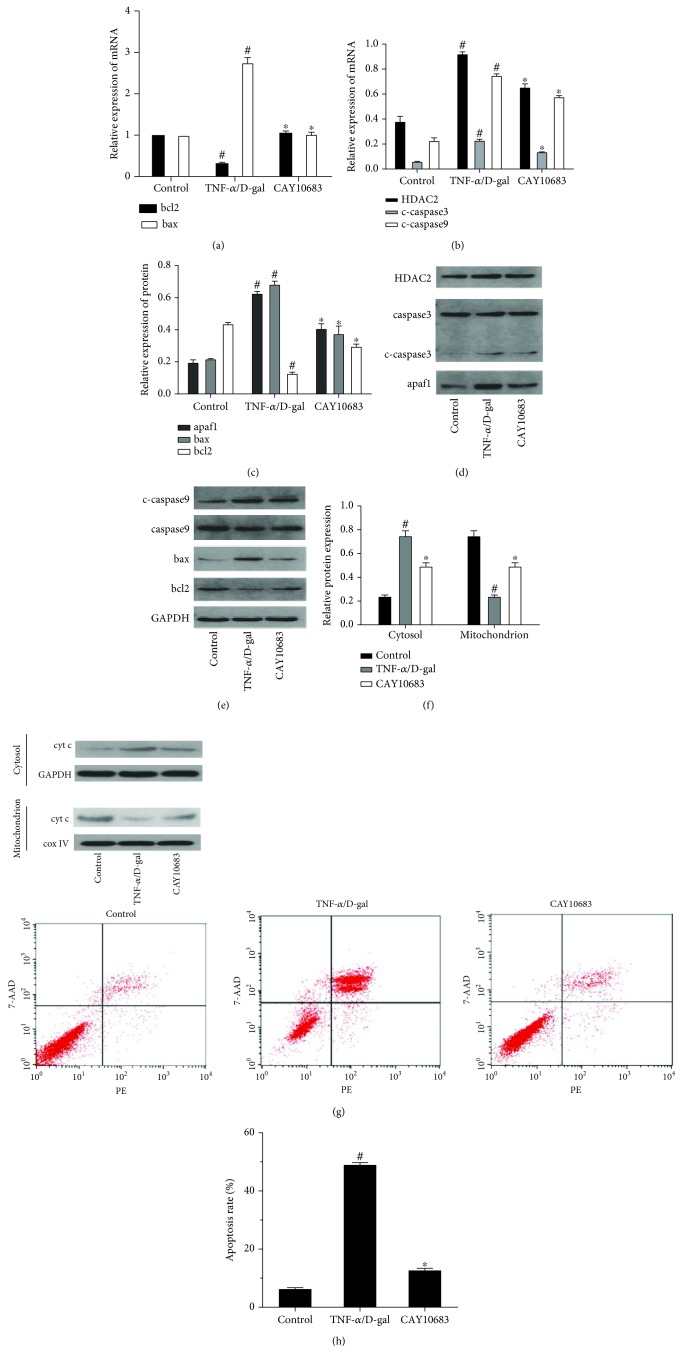
CAY10683 inhibited the mitochondrial apoptotic pathway in the TNF-*α*/D-gal-treated LO2 cells. (a) The mRNA level of bcl2 and bax mRNA levels were detected by RT-PCR. (b-g) The protein expressions of HDAC2, cyt c in mitochondrion, cyt c in cytosol, caspase 3, c-caspase 3, caspase 9, c-caspase 9, apaf1, bcl2, and bax were detected by western blot. (h) The staining with Annexin V-phycoerythrin/7-aminoactinomycin D (Annexin V-PE/7AAD) and flow cytometry were performed to detect the influence LO2 cell apoptosis. ^#^*P* < 0.05, compared with the control group. ^∗^*P* < 0.05, compared with the TNF-*α*/D-gal-treated model group. c-caspase3: cleaved-caspase 3, c-caspase9: cleaved-caspase 9.

Compared with the control group, the bcl2 mRNA level was significantly decreased and the bax mRNA level was significantly increased in the model cell group (*P* < 0.05). CAY10683 treatment could significantly increase bcl2 and decrease bax mRNA levels (*P* < 0.05) (Figure 1(a)). As shown in Figures 1(b)–1(g), the expressions of HDAC2, cyt c in cytosol, cleaved-caspase 3, cleaved-caspase 9, apaf1, and bax were significantly elevated, while the expressions of bcl2 and cyt c in the mitochondrion were significantly suppressed in the model group (*P* < 0.05). CAY10683 treatment significantly decreased HDAC2, cyt c in cytosol, cleaved-caspase 3, cleaved-caspase 9, apaf1 and bax protein levels, and elevated bcl2 and cyt c in mitochondrion protein levels (*P* < 0.05). There was no difference between the protein expressions of caspase 3 and caspase 9. Compared with the model group, the apoptosis rate in the CAY10683 group was significantly decreased (Figure 1(h), *P* < 0.05).

### 3.2. The Levels of Mitochondrial Apoptosis Signaling Molecules in LO2 Cells Were Changed after HDAC2 Knockdown or Overexpression

**Figure 2:**
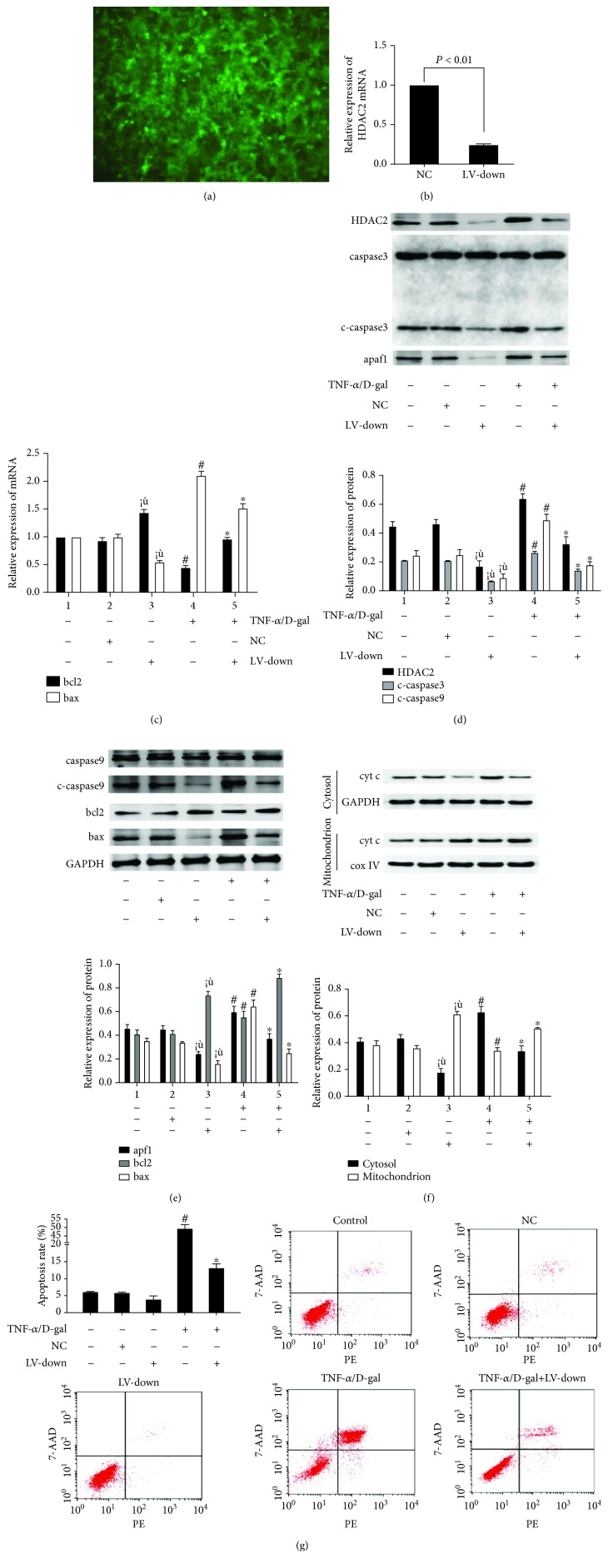
The levels of mitochondrial apoptosis signaling molecules in LO2 cells were decreased after HDAC2 knockdown. (a) The HDAC2 knockdown lentiviral vector was made and transfected into LO2 cells. GFP was observed with a fluorescence microscope after 72 h. (b) At 72 h after transfection, the HDAC2 mRNA level was detected by RT-PCR. (c) The mRNA level of bcl2 and bax mRNA levels were detected by RT-PCR. (d-f) The protein expressions of HDAC2, cyt c in mitochondrion, cyt c in cytosol, caspase 3, c-caspase 3, caspase 9, c-caspase 9, apaf1, bcl2, and bax were detected by western blot. (g) The staining with Annexin V-PE/7AAD and flow cytometry were performed to detect the influence LO2 cell apoptosis. ^※^*P* < 0.05, compared with the control group. ^#^*P* < 0.05, compared with the control group. ^∗^*P* < 0.05, compared with the TNF-*α*/D-gal-treated model group.

**Figure 3:**
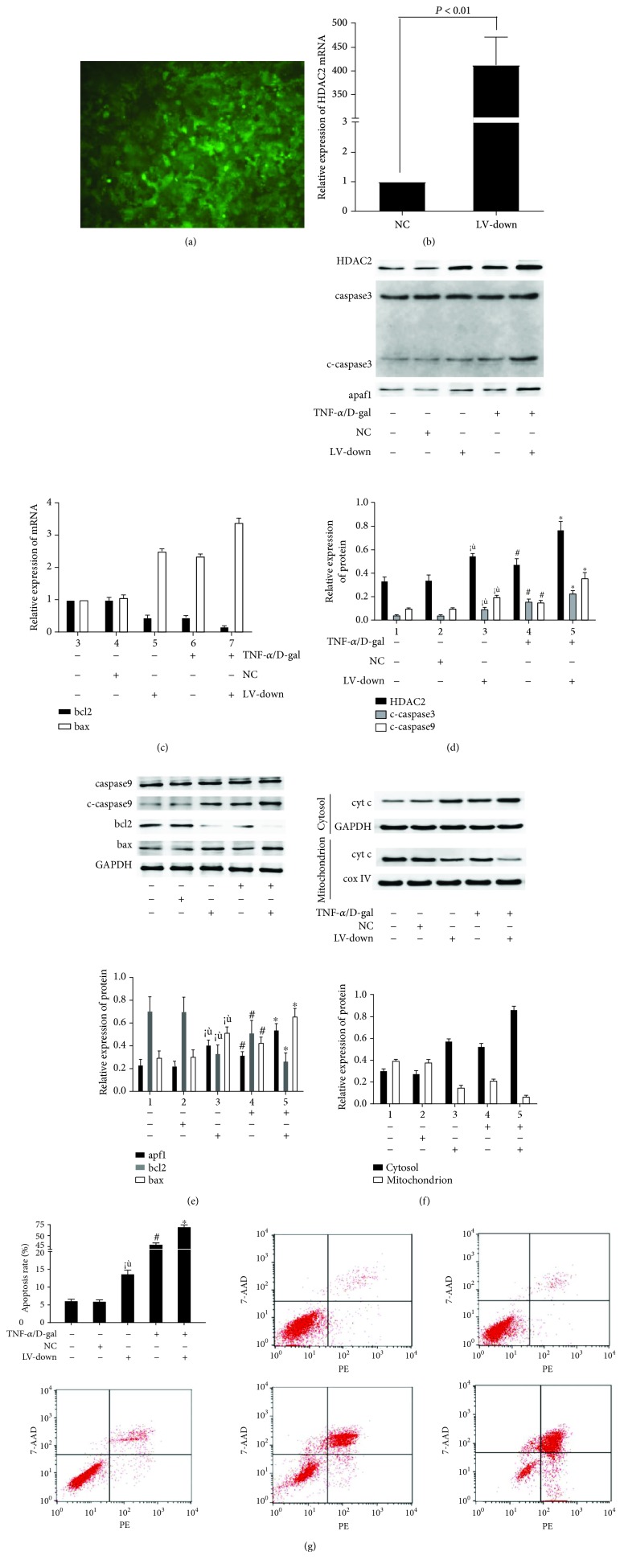
The levels of mitochondrial apoptosis signaling molecules in LO2 cells were increased after HDAC2 overexpression. (a) The HDAC2 overexpression lentiviral vector was made and transfected into LO2 cells. GFP was observed with a fluorescence microscope after 72 h. (b) At 72 h after transfection, the HDAC2 mRNA level was detected by RT-PCR. (c) The mRNA level of bcl2 and bax mRNA levels were detected by RT-PCR. (d-f) The protein expressions of HDAC2, cyt c in mitochondrion, cyt c in cytosol, caspase 3, c-caspase 3, caspase 9, c-caspase 9, apaf1, bcl2, and bax were detected by western blot. (g) The staining with Annexin V-PE/7AAD and flow cytometry were performed to detect the influence LO2 cell apoptosis. ^※^*P* < 0.05, compared with the control group. ^#^*P* < 0.05, compared with the control group. ^∗^*P* < 0.05, compared with the TNF-*α*/D-gal treated model group.

The HDAC2 knockdown or overexpression lentiviral vector (LV) was made and transfected into LO2 cells. Green fluorescent protein (GFP) was observed with a fluorescence microscope after 72 h (Figure 2(a) and Figure 3(a)). At 72 h after transfection, the HDAC2 mRNA level was obviously decreased in the LV-down group (Figure 2(b), *P* < 0.05). The interactions among the molecules of mitochondrial apoptosis signaling were observed. There was no significant difference between the control and lentivirus negative control (NC) groups for bcl2 and bax mRNA levels (Figure 2(c)). The protein levels of HDAC2, cyt c in mitochondrion, cyt c in cytosol, cleaved-caspase 3, cleaved-caspase 9, apaf1, bcl2, and bax were decreased (Figures 2(d)–2(f)), and the apoptosis rate was decreased (Figure 2(g)). Moreover, in HDAC2 knockdown cells, the bax mRNA level was decreased, and the bcl2 mRNA level was increased; compared with the control group, there was a significant difference (*P* < 0.05, Figure 2(c)). The protein expressions of HDAC2, cyt c in cytosol, cleaved-caspase 3, cleaved-caspase 9, apaf1, and bax were decreased; the levels of bcl2 and cyt c in the mitochondrion were increased in the LV-down group (Figures 2(d)–2(f)).

HDAC2 knockdown cells were treated with TNF-*α*/D-gal for 24 h. Compared with the model group, the bax mRNA level in the LV-down/TNF-*α*/D-gal group was decreased (*P* < 0.05), and the bcl2 mRNA level was increased (*P* < 0.05, Figure 2(c)). The protein expressions of HDAC2, cyt c in cytosol, cleaved-caspase 3, cleaved-caspase 9, apaf1, and bax were decreased, and the levels of bcl2 and cyt c in the mitochondrion were decreased (*P* < 0.05, Figures 2(d)–2(f)). The apoptosis rate was also decreased in the LV-down/TNF-*α*/D-gal group, compared with the model group (*P* < 0.05, Figure 2(g)), whereas the levels of mitochondrial apoptosis signaling molecules showed the opposite change after HDAC2 overexpression (Figures 3(b)–3(f)).

### 3.3. The Level of Histones H3 Deacetylated by Histone Deacetylase 2 in Different LO2 Cell Groups

**Figure 4:**
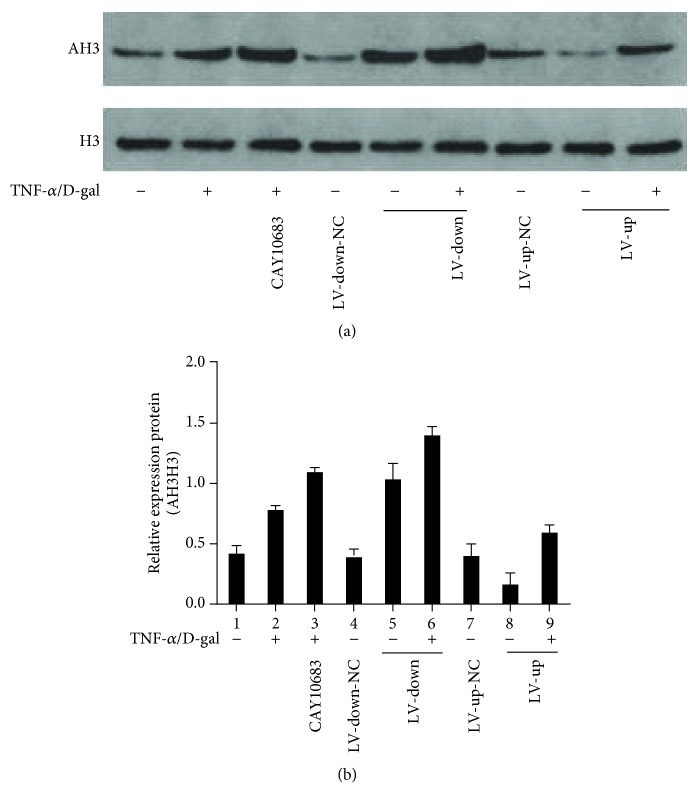
The level of histone H3 deacetylated by histone deacetylase 2 in different LO2 cell groups. ^※^*P* < 0.05, compared with the NC group. ^#^*P* < 0.05, compared with the control group. ^∗^*P* < 0.05, compared with the TNF-*α*/D-gal treated model group. NC: normal control.

As shown in Figures 4(a) and 4(b), the AH3 protein level was increased in the model group, compared with the control group (*P* < 0.05). CAY10683 could significantly enhance AH3 protein expression, compared with the model group (*P* < 0.05). However, AH3 was upregulated or downregulated in the HDAC2-LV-down or HDAC2-LV-up group, respectively (*P* < 0.05). It was suggested that TNF-*α*/D-Gal could increase the AH3 protein level in either the HDAC2-LV-down or the HDAC2-LV-up group (*P* < 0.05).

### 3.4. CAY10683 Could Alleviate the Hepatic Pathology and Liver Function in ALF Rats

**Figure 5:**
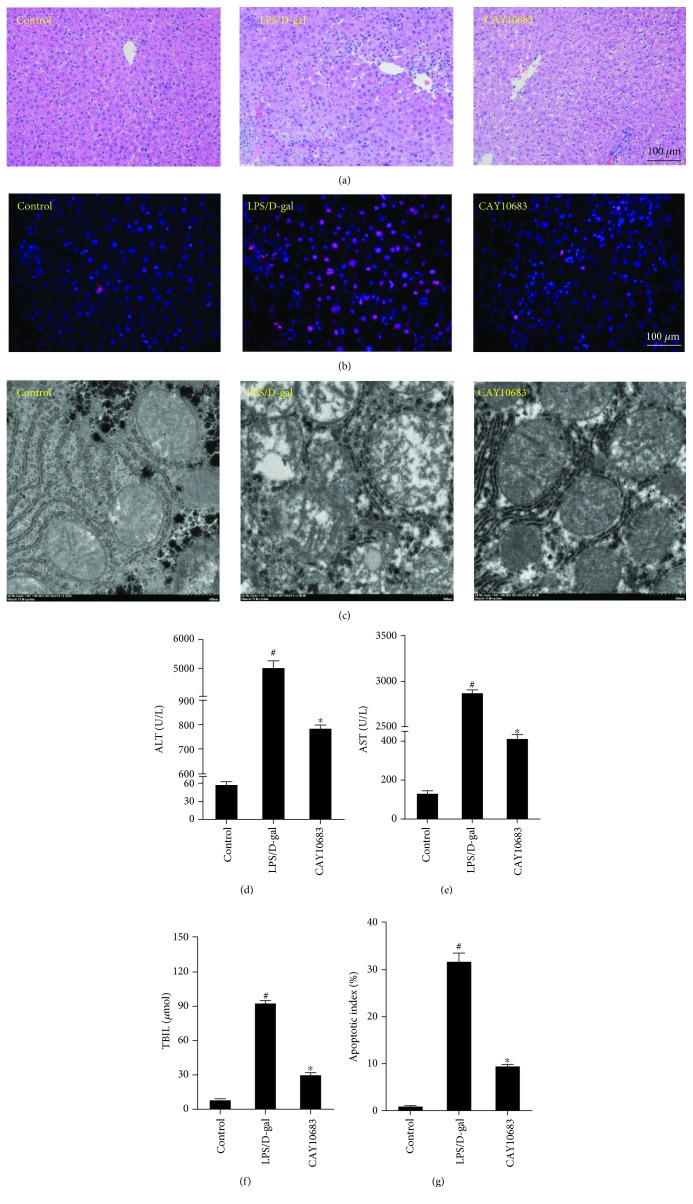
CAY10683 could alleviate the hepatic pathology and liver function in ALF rats. (a) HE staining was used to detect pathological changes in liver tissue. (b, f) Tunnel staining was used to detect the apoptotic index in liver tissue. (c) The ultrastructure of liver tissue and the mitochondrial membranes were detected by electron microscopy. (d-f) The rat serum ALT, AST, and TBIL levels were detected by the fully automated Aeroset chemistry analyzer. ^#^*P* < 0.05, compared with the control group. ^∗^*P* < 0.05, compared with the LPS/D-gal-treated model group.

As in HE staining shown in Figure 5(a), the structure of liver lobules in the control group was clear. The arrangement of liver cells was neat, and the infiltration of inflammatory cells was not observed around the liver cells. In the rat model group, the liver lobular structure was unclear, and hepatocytes were necrotic surrounded by inflammatory cell infiltration. However, the hepatic lobule structure in CAY10683 treatment rat liver was clearer than that in the model group, and the infiltration of inflammatory cells was also reduced. As shown in Figure 5(b) and Figure 5(g), tunnel staining presented that the apoptotic index in the CAY10683 treatment group was distinctly decreased, when compared with the model group (*P* < 0.05). For the ultrastructure of liver tissues (Figure 5(c)), in the control group, the mitochondrial inner and outer membranes were intact, the mitochondrial raft structure was clear, and the continuity was complete. In the model group, the swollen mitochondria in hepatocytes were found, the continuity of the inner and outer membranes was obviously destroyed, the mitochondrial cristae were broken, and the structure was fuzzy. However, in the CAY10683 treatment group, the swollen mitochondria in hepatocytes were significantly less than those in the model group. The membrane continuity was more complete, the mitochondrial membrane rupture was less, and the structure was clearer in the CAY10683 group than that in the model group. In addition, as shown in Figures 5(e)–5(g), the serum ALT, AST, and TBIL levels significantly decreased in the CAY10683 treatment group compared with the model group (*P* < 0.05).

### 3.5. CAY10683 Alleviates Liver Damage through Regulating the Mitochondrial Apoptotic Pathway in ALF Rats

**Figure 6:**
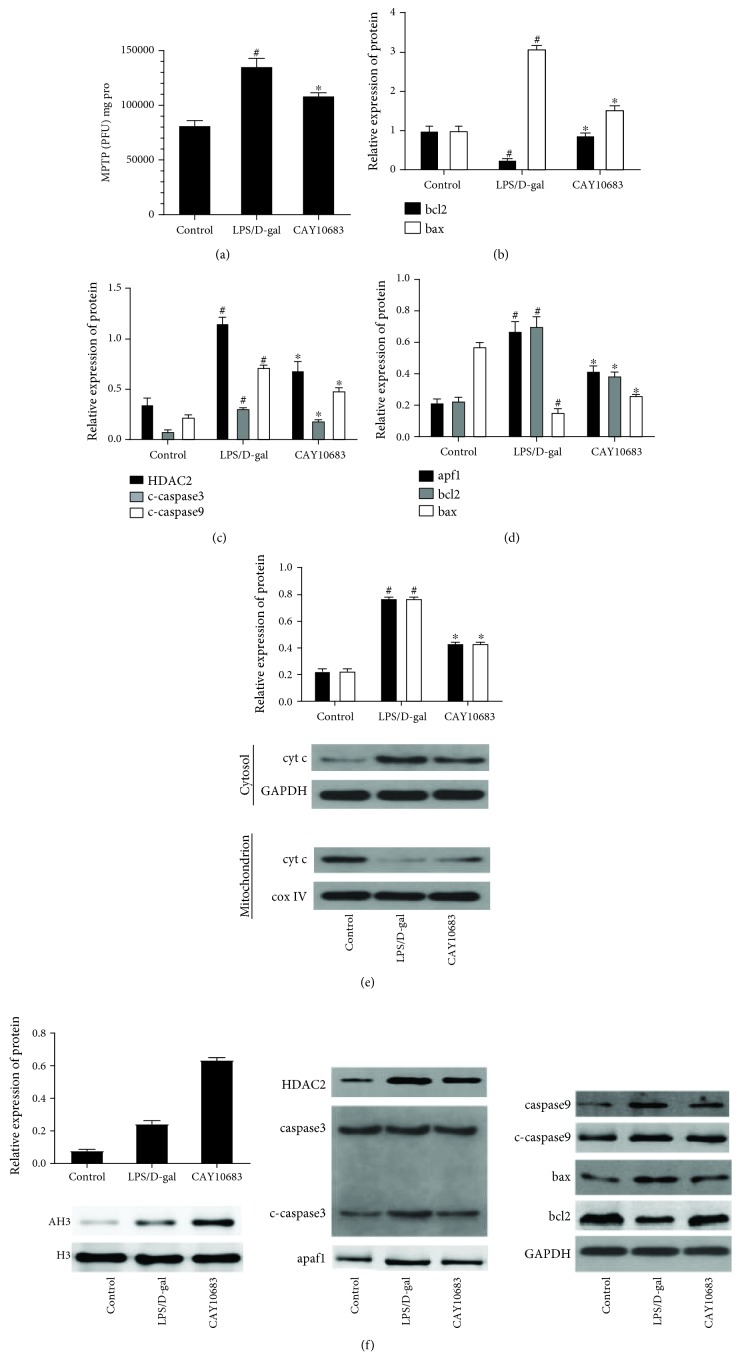
CAY10683 alleviates liver damage through regulating the mitochondrial apoptotic pathway in ALF rats. (a) The RFU value was detected by the purified MPTP fluorescence detection kit. (b) The mRNA level of bcl2 and bax mRNA levels were detected by RT-PCR. (c-e) The protein expressions of HDAC2, cyt c in mitochondrion, cyt c in cytosol, caspase 3, c-caspase 3, caspase 9, c-caspase 9, apaf1, bcl2, and bax were detected by western blot. (f) The protein expressions of AH3 and H3 were detected by western blot. ^#^*P* < 0.05, compared with the control group. ^∗^*P* < 0.05, compared with the LPS/D-gal-treated model group.

As shown in Figure 6(a), the RFU value was increased in the CAY10683 treatment group, compared with the model group (*P* < 0.05). The bcl2 mRNA level in the model group was significantly decreased, and the bax mRNA level was significantly increased, compared with the control group (*P* < 0.05, Figure 6(b)). CAY10683 treatment significantly increased the bcl2 mRNA level and decreased the bax mRNA level, compared with the model group (*P* < 0.05). As shown in Figures 6(c)–6(e), the protein levels of HDAC2, cyt c in cytosol, cleaved-caspase 3, cleaved-caspase 9, apaf1, and bax were significantly elevated. The protein levels of bcl2 and cyt c in the mitochondrion were significantly suppressed in the model group (*P* < 0.05). CAY10683 treatment significantly decreased the protein levels of HDAC2, cyt c in cytosol, cleaved-caspase 3, cleaved-caspase 9, apaf1, and bax and elevated bcl2 and cyt c in the mitochondrion (*P* < 0.05). There was no significant difference between the protein expressions of caspase 3 and caspase 9. As shown in Figure 6(f), the AH3 protein level was increased in the model group, compared with that in the control group (*P* < 0.05). CAY10683 could significantly elevate the AH3 protein level, compared with the model group (*P* < 0.05).

## 4. Discussion

Regarding the pathogenesis of acute liver failure, the “two-hit” hypothesis is widely recognized. The primary injury is induced by the virus directly or indirectly (immune responses). The secondary injury is caused by the release of major proinflammatory cytokines in the gut-liver axis [20]. It is currently recognized that endotoxemia and hepatocyte apoptosis might be the most important factors, which have an extreme impact on the occurrence and development of liver disease. Previous studies have shown that the patients with liver failure had severe intestinal microecological imbalances. The overgrowing intestinal bacteria could decrease intestinal colonization resistance and destroy intestinal wall barrier function, which resulted in intestinal bacteria (including endotoxin and intestinal cytokines) translocation [21]. As a major component of endotoxin, LPS induced localized nonspecific hypersensitivity reactions in the liver. It further causes liver microcirculation disturbance and leads to severe damage of liver cells. Endotoxin could also stimulate liver Kupffer cells to release TNF-*α*, IL-1*β*, IL-6, and other cytokines, which could cause apoptosis and necrosis of hepatocytes [22]. LPS and/or TNF-*α* is involved in the vicious circle to destroy the hepatocytes, which may result in liver failure. Therefore, it is suggested that inflammation control and prevention of endotoxemia could be the key strategy for the treatment of ALF through reducing “two-hit.”

Hepatocyte apoptosis is one of the important pathogeneses of ALF. It is mainly mediated by the death receptor pathway and mitochondrial apoptosis pathway [23]. The mitochondrial pathway is the central circle of hepatocyte apoptosis. Hepatocyte apoptosis is mainly regulated by bcl2 family proteins, including the antiapoptotic molecules such as bcl-2 and bcl-xL, and proapoptotic molecules such as bmf, bid, bax, and bak [24]. Some proapoptotic molecules such as bid, bad, and bim could bind to other proapoptotic molecules, which express in the outer surface of the mitochondria or cytoplasm, such as bax and bak, resulting in the oligomerization of the proapoptotic molecules. Subsequently, the oligomeric molecules could insert into the mitochondrial membrane, which cause the increase in mitochondrial membrane permeability and the loss of transmembrane potential. These mitochondrial changes could further induce the releases of cyt c from the mitochondria into the cytosol. Then, cyt c binds to apaf1 in the presence of dATP to form a multimer, which is able to recruit caspase 9 precursors in the cytoplasm and promote the binding of caspase 9 to apoptotic bodies through the caspase recruitment domain (CARD) of the amino terminus of apaf1. The activated caspase 9 could further activate caspase 3, which would trigger a caspase cascade and induce apoptosis [25]. Caspase 3 is the most important apoptotic executor in the caspase family. It is responsible for the activation or inactivation of the key molecules, such as GSDME (gasdermin E) [26]. In addition, apoptosis-inducing factors (AIF) could be released from the mitochondria. It is involved in the activation of caspase, the release of cyt c, and the formation of apoptotic bodies. In the mitochondrial pathway, the increased expression of bcl2 could exert an antiapoptotic effect by reducing mitochondrial membrane permeability, inhibiting mitochondrial depolarization, and releasing cyt c [27, 28]. Bax could promote the release of cyt c and regulate cell apoptosis [29]. The antiapoptotic effect of bcl2 and the proapoptotic effect of bax have been widely recognized. Therefore, the ratio of bcl2/bax could reflect the apoptosis state. Increasing the ratio of bcl2/bax could be an effective way to inhibiting apoptosis [30].

It has been widely demonstrated that inhibiting the activity of some HDAC molecules could alleviate liver failure. Our previous study showed that HDAC broad-spectrum inhibitor trichostatin A (TSA) could effectively suppress the release of some inflammatory factors, improve liver and small intestine injury, increase the survival rate, and provide a protective effect in ALF or ACLF rats [31]. Another specific histone deacetylase 6 inhibitor, CY-1215, also known as ricolinostat, has reported many other effects such as anti-cancer [32], improvement of pulmonary fibrosis [33], and alleviation of peripheral neuropathy [34]. Our previous study has also demonstrated that ACY-1215 could inhibit the inflammatory response through the TLR4-MAPK/NF-*κ*B pathway in ALF rats [35]. A clinical study has shown that the activities of HDAC1 and HDAC2 are obviously elevated in the patients with chronic hepatitis B, especially in the patients with liver failure. The downregulated HDAC1 could reduce the production of inflammatory factors [36]. However, whether the HDAC inhibitor (HDACi) plays an anti-hepatocyte apoptosis role in liver failure is still kept unknown at present. Many evidences have demonstrated that HDACi can inhibit the apoptosis in some tissue parenchymal cells or functional cells apart from cancer cells. It has been shown that TSA could attenuate apoptosis during acute lung injury [37]. Similarly, HDAC broad-spectrum inhibitor sodium butyrate could improve cardiac function and suppress myocardial remodeling in diabetic mice through decreasing the active caspase 3 protein level and the amounts of apoptosis myocardial cells [38]. Another HDAC broad-spectrum inhibitor, sulforaphane (SFN), significantly inhibits the apoptosis of LPS-induced porcine monocyte-derived dendritic cells [39].

In the present study, a specific HDAC2 inhibitor CAY10683 was used in an in vitro study. CAY10683 treatment significantly increased the levels of bcl2 and cyt c in the mitochondrion and decreased the levels of HDAC2, cyt c in cytosol, cleaved-caspase 3, cleaved-caspase 9, apaf1, and bax in the cells of the model group. The apoptosis rate in the CAY10683-treated group was significantly decreased. In order to further observe the effects of modulations of HDAC2, HDAC2 knockdown and overexpression by lentiviral vector in LO2 cells were performed, respectively. These transfected cells were treated with TNF-*α*/D-gal for 24 h. Compared with the control group, both mitochondrial apoptosis pathway and apoptosis rate were inhibited in the LV-down group and were increased in the LV-up group.

In in vivo experiments of this study, CAY10683 could significantly improve hepatic pathology and biochemical function in ALF rats. Tunnel staining showed that the apoptotic index was distinctly decreased in the CAY10683 treatment group, when compared with model group rats. As shown in the ultrastructure of liver tissues, the swollen mitochondria in hepatocytes were significantly alleviated in the CAY10683 treatment group than those in ALF rats. The mitochondrial crista continuity was complete, and the mitochondrial membrane rupture was also significantly reduced in the CAY10683 treatment group. The RFU value was increased in the CAY10683 treatment group when compared with the LPS/D-gal treated group. Likewise, the mitochondrial apoptosis pathway was activated in the model rat group and inhibited in the CAY10683 treatment group.

Previous studies have proved that HDAC2 can affect the histone level and dissociate histone H3 from the DNA molecule, thereby reducing the expression of the DNA molecule [40, 41]. In this study, we studied the mechanism of the HDAC2 level affecting apoptosis through regulating acetylated histone H3. When compared with the control group, the level of AH3 was increased in the TNF-*α*/D-Gal group. CAY10683 could significantly enhance the AH3 protein level compared with the TNF-*α*/D-Gal group without CAY10683. Likewise, in the HDAC2-LV-down or HDAC2-LV-up group, the AH3 protein level was upregulated or downregulated, respectively. Either in the HDAC2-LV-down or HDAC2-LV-up group, TNF-*α*/D-Gal could increase the AH3 protein level.

In fact, this preliminary study only demonstrated that inhibition of HDAC2 expression could alleviate apoptosis in acute liver failure. The mitochondrial apoptosis pathway included proapoptosis molecules (such as BMF, bid, bax, and bak) and antiapoptosis molecules (such as bcl-2 and bcl-xl). The mechanisms of modulations of HDAC2 effects on the specific antiapoptotic and/or proapoptotic molecules are still unknown. In addition, whether HDAC2 deacetylates histone H3 on some key molecule promoters, such as miR-155 [12], consequently affecting the apoptosis, needs to be further studied. In conclusion, modulations of histone deacetylase 2 via the mitochondrial apoptosis pathway offer a protective effect in acute liver failure. It is suggested that inhibiting the expression of HDAC2 could be a therapeutic agent for treating ALF.

## Figures and Tables

**Table 1:** Primers for RT-PCR.

Genes	Forward (5′-3′)	Reverse (5′-3′)
HDAC2 (human)	GCTACTACTACGACGGTGATATTGG	TTCTTCGGCAGTGGCTTTATGG
bcl2 (human)	CTGCACCTGACGCCCTTCACC	CACATGACCCCACCGAACTCAAAGA
bcl2 (rat)	GAGCGTCAACAGGGAGATGT	CAGCCAGGAGAAATCAAACAG
bax (human)	CGAGTGGCAGCTGACATGTTTT	TGAGGCAGGTGAATCGCTTG
bax (rat)	GAGCGAGTGTCTCCGGCGAATT	GCCACAAAGATGGTCACTGTCTG
GAPDH (human)	ACCACAGTCCATGCCATCAC	TCCACCACCCTGTTGCTGTA
GAPDH (rat)	GGCACAGTCAAGGCTGAGAATG	ATGGTGGTGAAGACGCCAGTA

## Data Availability

The histological examination, electron microscopy examination, biochemical index detection, PCR data, and WB data used to support the findings of this study are included within the article. There is no restriction on data access.
